# LNA effects on DNA binding and conformation: from single strand to duplex and triplex structures

**DOI:** 10.1038/s41598-017-09147-8

**Published:** 2017-09-08

**Authors:** Y. Vladimir Pabon-Martinez, You Xu, Alessandra Villa, Karin E. Lundin, Sylvain Geny, Chi-Hung Nguyen, Erik B. Pedersen, Per T. Jørgensen, Jesper Wengel, Lennart Nilsson, C. I. Edvard Smith, Rula Zain

**Affiliations:** 1 Department of Laboratory Medicine, Clinical Research Center, Karolinska Institutet, SE-141 86 Huddinge, Stockholm Sweden; 2 Department of Biosciences and Nutrition, Karolinska Institutet, SE-141 83 Huddinge, Sweden; 3 Institut Curie, PSL Research University, UMR 9187-U 1196, CNRS-Institut Curie, INSERM, Centre Universitaire, Orsay, France; 4 Department of Physics, Chemistry and Pharmacy, Nucleic Acid Center, University of Southern Denmark, DK-5230 Odense M, Denmark; 5 Department of Clinical Genetics, Centre for Rare Diseases, Karolinska University Hospital, SE-171 76 Stockholm, Sweden

## Abstract

The anti-gene strategy is based on sequence-specific recognition of double-strand DNA by triplex forming (TFOs) or DNA strand invading oligonucleotides to modulate gene expression. To be efficient, the oligonucleotides (ONs) should target DNA selectively, with high affinity. Here we combined hybridization analysis and electrophoretic mobility shift assay with molecular dynamics (MD) simulations to better understand the underlying structural features of modified ONs in stabilizing duplex- and triplex structures. Particularly, we investigated the role played by the position and number of locked nucleic acid (LNA) substitutions in the ON when targeting a c-*MYC* or *FXN* (*Frataxin*) sequence. We found that LNA-containing single strand TFOs are conformationally pre-organized for major groove binding. Reduced content of LNA at consecutive positions at the 3′-end of a TFO destabilizes the triplex structure, whereas the presence of Twisted Intercalating Nucleic Acid (TINA) at the 3′-end of the TFO increases the rate and extent of triplex formation. A triplex-specific intercalating benzoquinoquinoxaline (BQQ) compound highly stabilizes LNA-containing triplex structures. Moreover, LNA-substitution in the duplex pyrimidine strand alters the double helix structure, affecting x-displacement, slide and twist favoring triplex formation through enhanced TFO major groove accommodation. Collectively, these findings should facilitate the design of potent anti-gene ONs.

## Introduction

Triple-helix (triplex) structures of DNA and RNA have emerged as potential regulators of biological activity, which has led to the revival of the anti-gene field^1^. Sequence-specific DNA recognition by an oligonucleotide (ON) forming a triplex structure has been largely exploited to regulate gene expression at the transcriptional level, and to direct modifications of genomic DNA at selected sites through mutagenesis or homologous recombination^2–4^. However, when compared to other nucleic acid-based approaches, there are some challenges facing TFO-targeting of double-strand DNA (dsDNA) such as ON binding affinity and stability of the triplex structure in a genomic context^5, 6^.

**Figure 1:**
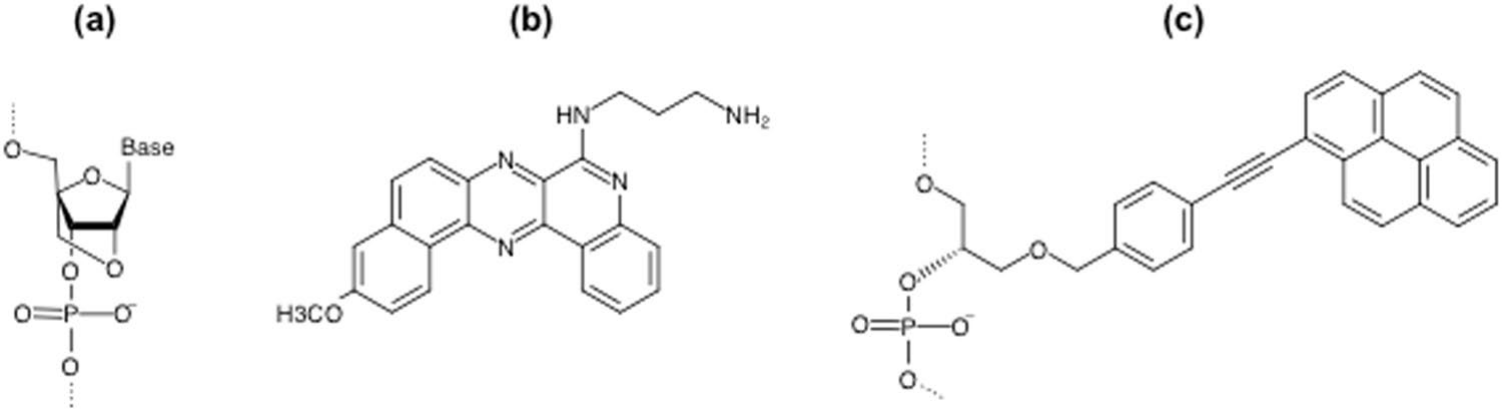
Chemical structures. (**a**) Locked nucleic acid (LNA). (**b**) Triplex specific DNA intercalating compound: Benzoquinoquinoxaline (BQQ). (**c**) DNA intercalator: p-Twisted intercalating nucleic acid (p-TINA).

According to the binding modes, anti-gene ONs are grouped as: (a) TFOs that bind to the polypurine strand in the major groove of dsDNA by Hoogsteen (HG) (parallel orientation) or reverse HG hydrogen bonds (antiparallel) between the bases forming a triplex structure^2, 3, 7–9^; (b) ONs that bind to one of the DNA strands by Watson-Crick (WC) hydrogen bonds leading to the displacement of the other strand. In the latter case a double-strand invasion (DSI) complex is efficiently formed by oligomers containing locked nucleic acid (LNA)^10, 11^ or peptide nucleic acid (PNA)^12–17^. LNA (Fig. 1a) is a synthetic nucleotide analogue characterized by a methylene bridging the 2′-oxygen and 4′-carbon of the ribose^18^. Fully substituted LNA ONs are less efficient in forming triplex structures^19^ and attempts have been made to set some rules for the design of LNA-based TFOs^20^. LNA and PNA oligomers include constructs with the capacity to simultaneously target dsDNA in both strands causing DSI^21–23^ or double duplex invasion^24^, respectively. LNA and PNA have also been used in clamp type ONs where two oligomers connected by a flexible linker target the same polypurine sequence through a dual binding mode including both WC and HG hydrogen bonds^12, 25–29^.

BisPNA was the first modified ON construct where two arms connected by a linker form a bis-type structure^28, 29^. Recently, a new construct based on LNA, called bisLNA, was developed with the capacity to strand-invade into supercoiled dsDNA under physiological salt and pH conditions forming a triplex structure^25, 26^. PNA and LNA differ in their chemical structure, where PNA has uncharged peptidic backbone and LNA has conformationally restricted but negatively charged sugar-phosphodiester backbone. Therefore, it is reasonable to assume that the mechanism and kinetics of binding of PNA *vs*. LNA may be different.

To obtain a deep insight on the influence of LNA substitution on the formation and the structural feature of DNA duplex and triplex structure, we investigated both triplex- and WC-forming single strand ONs employing a combined approach of binding experiments and molecular dynamics (MD) simulations. Nucleic acid simulations using state-of-art force fields have been shown to be powerful tools to supply information to experiments, and to make rational prediction of structural and thermodynamic properties (see refs 30–32). MD simulations have previously been successfully used to investigate TFO binding to DNA duplexes both in parallel and antiparallel fashion^33–35^. Here we chose to target a polypurine • polypyrimidine DNA sequence derived from the nuclease hypersensitivity element of the c-*MYC* gene promoter^36^, as this proto-oncogene has been previously examined for TFO targeting *in vitro* ^37^ and in cell culture^38^.

We tested a series of different LNA-based ONs to examine the effect of position and number of LNA substitutions on the conformation of the single strand as well as the corresponding duplex and triplex structures. We also examined TFO conjugation of a DNA intercalating compound (twisted intercalating nucleic acid, TINA) focusing on its position within the TFO sequence. TINA is a flexible base-stacking monomer that has been shown to stabilize intermolecular triplex structures^39^ (Fig. 1c). Moreover, LNA-ONs were further employed to follow DSI and triplex formation as two parallel events aiming to reveal the molecular mechanism of the dual mode of binding (WC and HG) of LNA-based clamp type constructs. All hybridizations were performed in intra-nuclear salt conditions, and in all cases a triplex-specific intercalating agent, Benzoquinoquinoxaline (BQQ) was used in parallel experiments to analyze triplex formation. BQQ is a pentacyclic aromatic compound (Fig. 1b), which intercalates specifically in triplex DNA with its aminopropyl side chain located in the minor groove, thereby discriminating between duplex and triplex structures^40, 41^. We found that LNA substituted ONs show conformation rearrangements, both in single and duplex strand states, which are beneficial for triplex formation, and the results were confirmed by binding experiments using electrophoretic mobility shift assay (EMSA). In addition, a clear effect on TFO binding to dsDNA is observed when LNA substitution takes part at the 3′-end of the ON in contrast to the 5′-end.

## Results and Discussion

### Conformational influences of LNA substitution in single strand TFO

**Figure 2:**
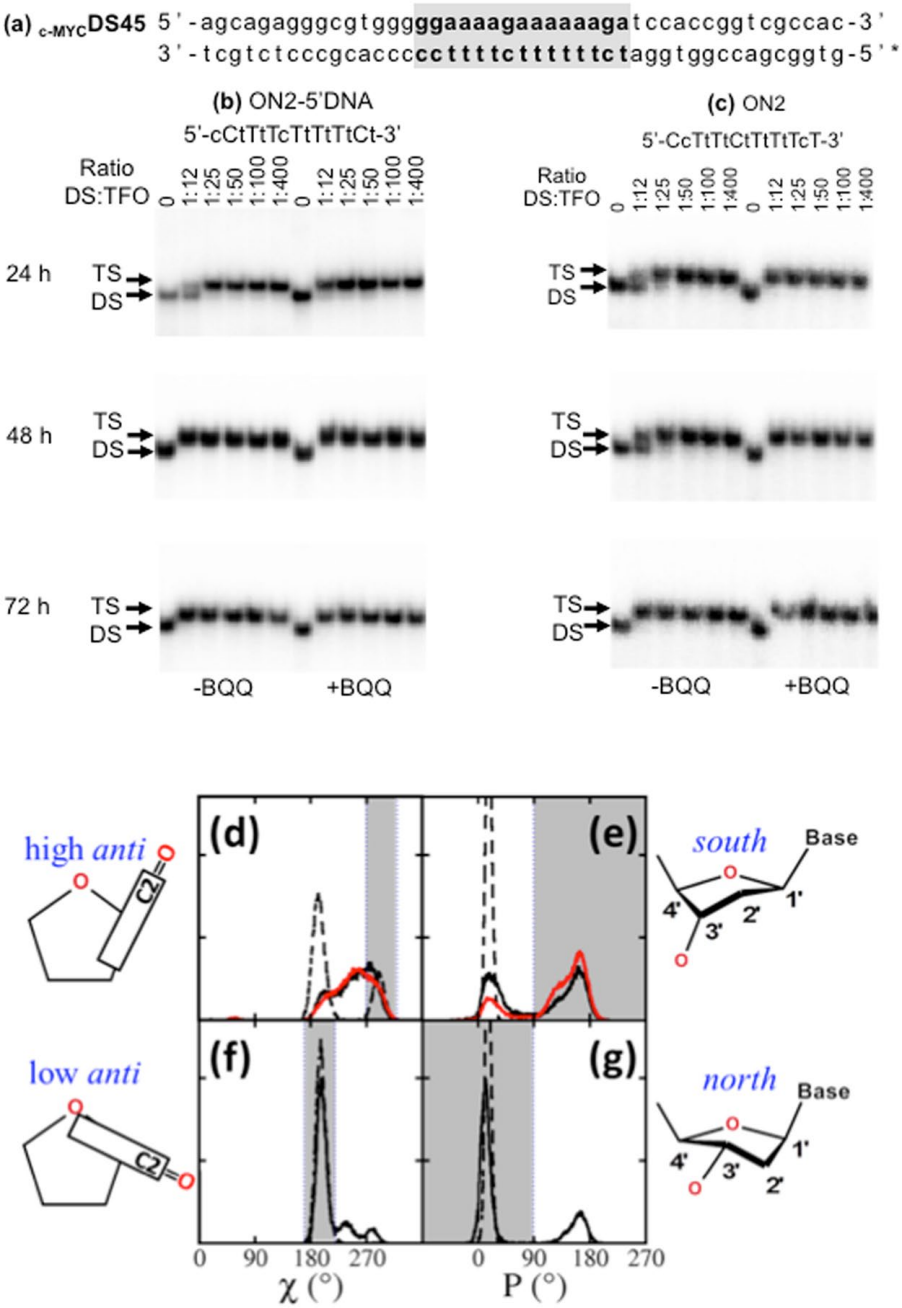
TFO binding of 15-mer ON sequences: (**a**) _c-MYC_DS45 and electrophoretic mobility shift profile of _c-MYC_DS45 in the presence of (**b**) ON2-5′DNA and (**c**) ON2. Hybridization with ON in the absence (left side) and in the presence (right side) of BQQ carried out during 24, 48 and 72 h. Triplex structures are detected as slower migrating bands. DNA duplex and triplex complexes are indicated as DS and TS, respectively. LNA is indicated in capital letters and DNA is in small letters. Distribution of glycosidic torsion (χ) and ribose pseudorotation (P): (Panels d and e) ON2-5′DNA (black) and ON1 (red) strands free in solution. (Panels f and g) ON2-5′DNA bound to the _c-__MYC_DS19. The sketches of base pair orientation and sugar pucker beside the graphs depict the conformation corresponding to the gray regions in the panel.

**Figure 3:**
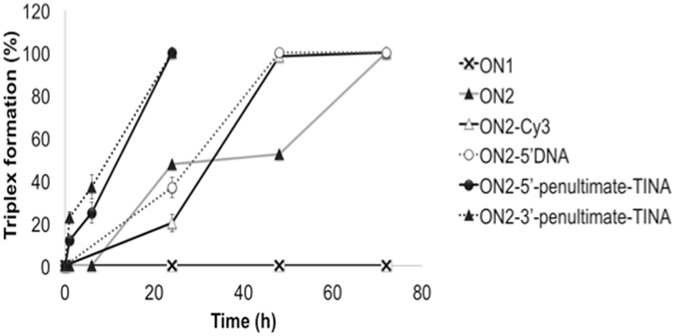
Comparison of triplex formation in the presence of different 15-mer LNA-ONs including TINA-ONs. Quantification of the amount of triplex formed using different TFO constructs (0.06 μM corresponding to 1:12 ratio of dsDNA:TFO). TFO binding was monitored during 0–72 h and analyzed using EMSA.

Initially, we examined binding of an LNA-modified (alternate LNA/DNA) ON (Table 1, ON2-5′DNA), to a dsDNA target (Fig. 2a) using increasing concentration of the ON and EMSA analysis. The ON concentration in relation to the dsDNA target is referred to as the dsDNA:TFO ratio throughout the text. Triplex formation was carried out in the presence or absence of a triplex-binding BQQ compound. Binding of ON2-5′DNA was clearly detected (Fig. 2b, 24 h) by the progressive increase in the intensity of the slower-migrating gel band corresponding to a triplex structure (TS) with increasing TFO concentration. In the absence of BQQ, 100% triplex formation was reached at 1:25 ratio of dsDNA:TFO (Fig. 2b), and in the presence of BQQ, ON2-5′DNA binding was completed at the lowest dsDNA:TFO ratio, demonstrating for the first time the ability of BQQ to intercalate and stabilize triplex structures formed by LNA-modified TFOs. On the other hand, an ON (15-mer) consisting of non-modified DNA failed to form triplex under these conditions even in the presence of BQQ; clearly demonstrating the enhanced hybridization capacity of LNA-based TFOs (Fig. 3 and Supplementary Fig. S1b).

**Table 1:** Oligonucleotide sequences studied.

Name	Length (nt)	Sequence
ON1	15	5′-ccttttcttttttct-3′
ON2	15	5′-CcTtTtCtTtTtTcT-3′
ON2-Cy3	15	5′-Cy3-cCtTtTcTtTtTtCt-3′
ON2-5′DNA	15	5′-cCtTtTcTtTtTtCt-3′
ON2-3′LNA • reduced	15	5′-CcTtTtCtTttttct-3′
ON2-5′LNA • reduced	15	5′-ccttttCtTtTtTcT-3′
ON2-5′-penultimate-TINA	15	5′-C**P**cTtTtCtTtTtTcT-3′
ON2-center-TINA	15	5′-CcTtTtC**P**tTtTtTcT-3′
ON2-3′-penultimate-TINA	15	5′-CcTtTtCtTtTtTc**P**T-3′
ON3	13	5′-CtTtTcTtTtTtC-3′
ON3-3′LNA • reduced	13	5′-CtTtTcTtTtttc-3′
ON3-5′LNA • reduced	13	5′-ctttTcTtTtTtC-3′
ON3-3′LNA • reduced-c > t	13	5′-TtTtTcTtTtttt-3′
ON3-5′LNA • reduced-c > t	13	5′-ttttTcTtTtTtT-3′
ON3-5′-penultimate-TINA	13	5′-C**P**tTtTcTtTtTtC-3′
ON3-3′-penultimate-TINA	13	5′-CtTtTcTtTtTt**P**C-3′
ON3-5′-ultimate-TINA	13	5′-**P**CtTtTcTtTtTtC-3′
ON3-3′-ultimate-TINA	13	5′-CtTtTcTtTtTtC**P**-3′
ON3-5′-3′-TINA	13	5′-**P**CtTtTcTtTtTtCP-3′
ON4-3′LNA • reduced	15	5′-CtTcTtCtTcttctt-3′
ON4-5′LNA • reduced	15	5′-cttcttCtTcTtCtT-3′
WC29	29	5′-Cy5-tCtTtTtTcTtTtCcCccAcgCccTctGc-3′
bisLNA49	49	5′-Cy3-CcTtTtCtTtTtTcT-tctct-tCtTtTtTcTtTtCcCccAcgCccTctGc-3′

LNA is indicated in capital letters and DNA is in small letters; Cy3 or Cy5 indicates the fluorophore used; **P**, p-TINA. ON3-3′LNA ⦁ reduced-c > t and ON3-5′LNA ⦁ reduced-c > t are sequences used for simulation where cytosine (c) was substituted by thymine (t). ON4-3′LNA ⦁ reduced and ON4-5′LNA ⦁ reduced are sequences used for simulation where the target sequence is derived from the *FXN* gene. The target sequence for all the other ONs is derived from the promoter of the c*-MYC* gene.

To understand the causal structural characteristics of LNA-based ONs, we compared the conformation of single strand ON1 (full DNA) and ON2-5′DNA using MD simulation. As shown in Fig. 2d and e, the LNA sugar pucker is fixed in *north*, whereas DNA sugar prefers *south* over *north*. The locked furanose also influences the base conformation. LNA bases are restricted to low and high *anti*, whereas DNA bases have a wider range of *anti* conformations. When DNA nucleotides are flanked by LNA nucleotides (as in ON2-5′DNA), we observed a slightly increased *north* conformation of the sugars, compared to ON1 (Fig. 2e). A similar conformational steering effect of LNA nucleotides on flanking DNA nucleotides has been shown by nuclear magnetic resonance (NMR) studies in a duplex context^42, 43^.

In the triplex model, the WC and HG base pairs are stable except for the spontaneous base pair opening between the end residues of ON2-5′DNA and duplex. The sugars in the WC-purine strand are all in *south* conformation whereas both *north* and *south* are present in the WC-pyrimidine strand and TFO (data not shown). Overall, the sugar and base triplet conformations in the simulated triplexes are consistent with the NMR structure of an analogous intramolecular triplex^44^.

When ON2-5′DNA is bound to the major groove of the DNA duplex, the DNA nucleotides in the TFO show more narrow distributions of both sugar pucker and glycosidic torsion, *i.e*. mainly in *north* and low *anti* respectively (Fig. 2f and g). The conformational rearrangement of LNA nucleotides is very small. Obviously, the sugar pucker and glycosidic torsion distribution of ON2-5′DNA in the single strand are more similar to those in the triplex compared to ON1 containing only DNA. This suggests that the pattern of alternating DNA/LNA in ON2-5′DNA, as compared to non-modified ON1, promotes a single strand conformation which facilitates binding to the major groove of duplex DNA with lower entropic cost. This is in agreement with previous reports regarding other 2′-*O*-modifications, which confer *north* conformation in the TFO and require less rearrangement of the single strand^45–47^. Taken together, our MD simulation results provide an explanation for the superior binding of LNA-based TFOs, in comparison to non-modified TFOs, as observed in the case of ON2-5′DNA and previously reported analogues^19, 20^.

### Effect of the number of LNAs and 3′ *vs*. 5′-end position in TFO

Sun *et al*. previously suggested a few rules for the design of LNA-TFOs^20^. It was then recommended to start the LNA substitution in a TFO at the 5′-end. To test this design we inverted the order of DNA and LNA nucleotides in ON2-5′DNA and evaluated the triplex forming efficiency of ON2 (Table 1). However, only 50% triplex was detected at a DNA:TFO ratio of 1:12 when ON2 binding was allowed to proceed during 48 hours (Figs 2c and 3), as compared to 100% triplex formation in the presence of ON2-5′DNA at the same time point (Figs 2b and 3). The results indicate that alternate substitution of DNA by LNA starting from the 5′-end of the examined TFO affects negatively its initial binding despite the fact that ON2 has a slightly higher LNA content (8 vs. 7 LNAs). On the other hand, the thermodynamic stability of the end complex is comparable for both TFOs, as judged from the results at 72 h of TFO binding (Figs 2b, c and 3).

**Figure 4:**
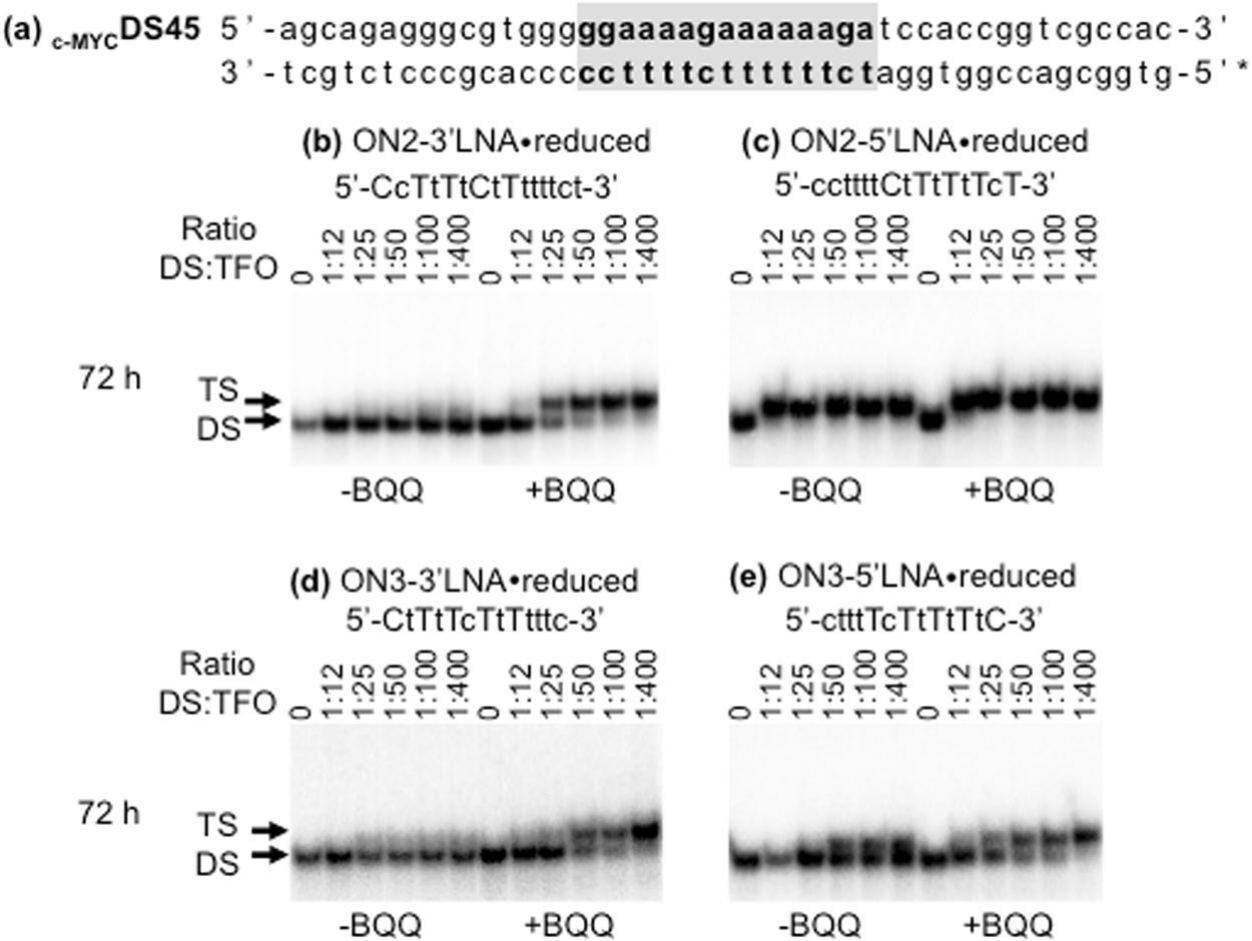
TFO binding of 13- and 15-mer ON sequences with different amounts of LNA substitutions at one of the ends. (**a**) _c-MYC_DS45, (**b**,**c**,**d**,**e**) Electrophoretic mobility shift profile of _c-MYC_DS45 in the presence of four different pyrimidine TFOs, as indicated. Hybridization with ON in the absence (left side) and in the presence (right side) of BQQ was carried out during 72 h. Triplex structures are detected as slower migrating bands. DNA duplex and triplex complexes are indicated as DS and TS, respectively. LNA is indicated in capital letters and DNA is in small letters.

Moreover, it has been shown that the total number of LNA modifications in a TFO has direct impact on dsDNA binding kinetics and triplex stability^19, 20, 48^. It is also known that both target and TFO sequence composition are reflected in triplex formation and stability^49^. Therefore, we decided to examine different variants of the same TFO sequence, where changes were introduced with regard to the LNA content and position. To examine if LNA content in TFOs is more significant at the 5′- or 3′-end, we designed an ON, which carries six consecutive unmodified nucleotides at the 3′-end (ON2-3′LNA ⦁ reduced) or 5′-end (ON2-5′LNA ⦁ reduced) of the 15-mer TFO (Table 1), and binding to the _c-MYC_DS45 dsDNA target (Fig. 4a) was carried out during 24, 48 (data not shown), and 72 h. EMSA analysis and quantification of the intensity of the gel bands indicate that a TFO with reduced LNA content at the 3′-end (Fig. 4b) is by far less efficient than a TFO with reduced LNA content at the 5′-end (Fig. 4c). As a matter of fact, we did not detect triplex formation in the presence of ON2-3′LNA ⦁ reduced after 72 h unless binding was performed in the presence of BQQ.

**Figure 5:**
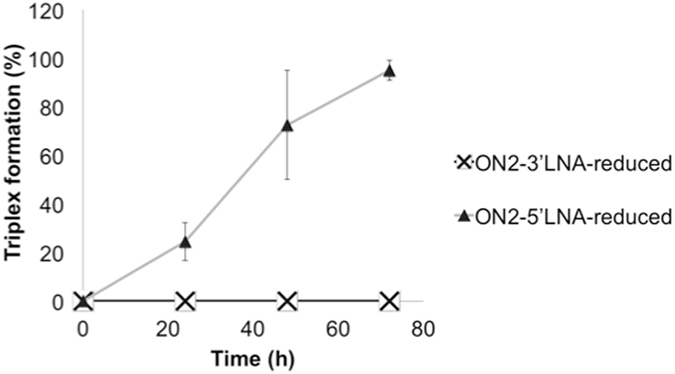
Comparison of triplex formation in the presence of 15-mer ONs with reduced LNA-content at the 3′- vs. 5′-end. Quantification of the amount of triplex formed using ON2-3′-LNA-reduced and ON2-5′-LNA-reduced (0.06 μM corresponding to 1:12 ratio of dsDNA:TFO). TFO binding was monitored during 0–72 h and analyzed using EMSA.

However, because these two sequences were not symmetrical in terms of the end-nucleotides, we considered examining a second set of TFOs, which were more “symmetrical” at the 3′- and 5′-ends, while avoiding the presence of two consecutive cytosines. The new TFO sequences (ON3-3′LNA ⦁ reduced and ON3-5′LNA ⦁ reduced) are 13-mers and contain a stretch of five LNAs of every second nucleotide at the one end and a stretch of four DNA nucleotides at the other end (Table 1, Fig. 4d and e). Consistently, our results show that substitution of LNA by DNA at the 3′-end of the TFO has stronger negative effect on triplex formation (Fig. 5). Previous studies have examined the effects of introducing a cluster of modified nucleotides at either end, or in the middle of a TFO. For example, substitution by a cluster of 3-4 2′-*O*-(2-aminoethyl) residues resulted in higher triplex bioactivity as compared to dispersed modifications, which is described as an effect of a decreased dissociation rate^50^. However, no significant difference was found when the sugar modification was placed at 3′ or 5′-end of the TFO^51^. On the other hand, introduction of cationic modifications at the 5′-end of TFO was described to be more efficient than the corresponding 3′-end modification^52^.

**Figure 6:**
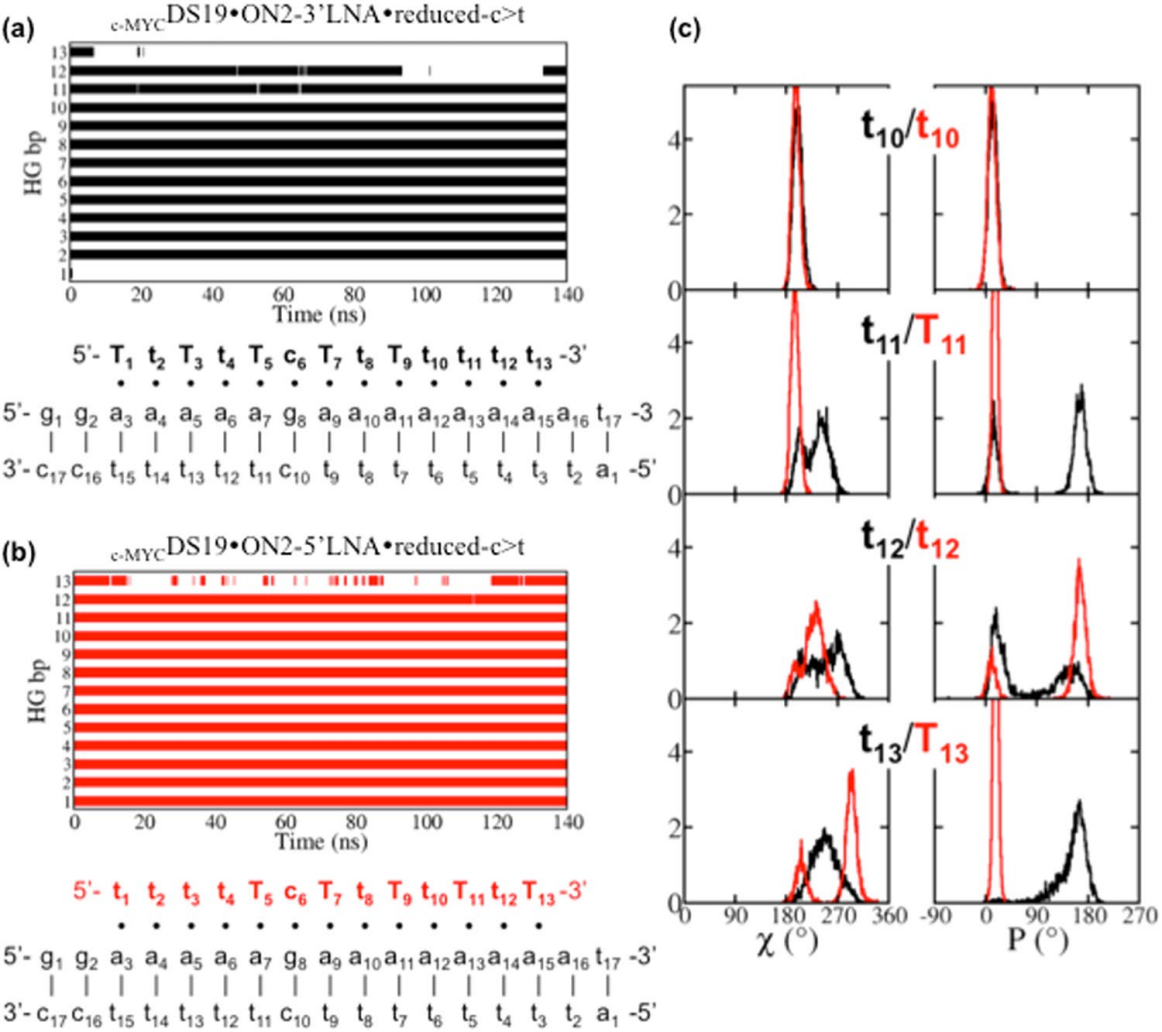
Effect of LNA position on TFO binding. HG hydrogen bonds as the function of time together with secondary structure sketch are shown. (Panel a) Triplex _c-MYC_DS19 ⦁ ON3-3′LNA ⦁ reduced-c > t and (Panel b) Triplex _c-MYC_DS19 ⦁ ON3-5′LNA ⦁ reduced-c > t; the blank spaces correspond to the loss of the hydrogen bonds. (Panel c) Distributions of χ and P for the last four nts at 3′-end in each TFO, ON3-3′LNA ⦁ reduced-c > t is in black and ON3-5′LNA ⦁ reduced-c > t in red.

We further examined our findings by simulation of the triplex formed in the presence of ON2-3′LNA ⦁ reduced or ON2-5′LNA ⦁ reduced, which showed consistent tendency. With respect to the hydrogen bond persistence of triplex base pairs as the function of simulation time, _c-MYC_DS19 ⦁ ON2-5′LNA ⦁ reduced lost two base pairs at the 3′-end whereas _c-MYC_DS19 ⦁ ON2-3′LNA ⦁ reduced lost three (Supplementary Fig. S2). However, an artifact structural disturbance on the duplex was observed for _c-MYC_DS19 ⦁ ON2-3′LNA ⦁ reduced: once the 5′-end protonated cytosine became unpaired from the HG partner it interacted with nearby duplex phosphate thus destabilizing the duplex. This is probably due to the fixed protonation state of the atomic model, whereas in reality the protonation is probably lost when the base pair is opened and formed again when the base pair is recovered. To eliminate this effect from the asymmetrical sequence, we performed the simulations with the two corresponding 13-mer TFOs where each of the end cytosines was substituted by thymine (_c-MYC_DS19 ⦁ ON3-3′LNA ⦁ reduced-c > t and _c-MYC_DS19 ⦁ ON3-5′LNA ⦁ reduced-c > t). Consequently, we found that _c-MYC_DS19 ⦁ ON3-5′LNA ⦁ reduced-c > t kept more base pairs than _c-MYC_DS19 ⦁ ON3-3′LNA ⦁ reduced-c > t and led to less structural deviation, and base pair opening is mainly observed in the 3′ position (Fig. 6a and b). Furthermore, we observed that DNA nucleotides near the 5′-end of _c-MYC_DS19 ⦁ ON3-5′LNA ⦁ reduced-c > t still kept low-*anti* and *north* conformation (Supplementary Fig. S3a), a conformation observed for TFO in triplex, whereas DNA nucleotides near the 3′-end of _c-MYC_DS19 ⦁ ON3-3′LNA ⦁ reduced-c > t did not (Fig. 6c). These results suggest that stabilization of 3′ end promotes higher TFO binding affinity. This is also in agreement with the experimental observation that the lack of LNAs in 3′-end of the TFO impairs triplex formation whereas the presence of DNA, and not LNA, nucleotides at the 5′-end has essentially no effect (Fig. 4d and e).

To assess if our results can have a broader application, we examined an additional dsDNA target sequence (Table 2, _FXN_DS19) and corresponding two TFOs (Table 1, ON4-3′LNA⦁reduced and ON4-5′LNA⦁reduced), which differ in their LNA content at the 3′- and 5′-end, respectively, in analogy with the ONs described in the previous section. The binding site of the new sequence, which is derived from the *Frataxin* (*FXN)* gene associated with Friedreich′s ataxia disease, has a repeated GAA motif in the purine strand^53^. TFO binding of the *FXN* repeat sequence has been previously shown to form a pyrimidine motif triplex^54^. Both systems _FXN_DS19 ⦁ ON4-3′LNA⦁reduced and _FXN_DS19 ⦁ ON4-5′LNA⦁reduced quickly lost the first HG base pair at the 5′-end, but the remaining base pairs near the 5′-end were stable. In agreement with the results of _c-MYC_DS19⦁ON3-5′LNA⦁reduced-c/t and _c-MYC_DS19⦁ON3-3′LNA⦁reduced-c/t the destabilization of HG base pairs was mainly observed at the 3′-end, and more so for ON4-3′LNA⦁reduced than for ON4-5′LNA⦁reduced (Supplementary Fig. S4a and b). Considering the ON conformation during the first 70 ns of the simulation, the LNA-reduced parts of each ON4 are not stably low *anti* and *north* (Supplementary Fig. S4c), and with respect to the end most DNA nucleotides (the last two), ON4-3′LNA⦁reduced almost lost this conformational feature. The observation is consistent with the results observed for ONs evaluated with the c-*MYC* target sequence discussed previously, which suggests that in absence of LNA, the base pair opening happens more frequently from the 3′-end.

**Table 2:** Target sequences used for experiments.

Name	Length (nt)	Sequence
_c-MYC_DS45	45	5′-agcagagggcgtggg**ggaaaagaaaaaaga**tccaccggtcgccac-3′
		3′-tcgtctcccgcaccc**ccttttcttttttct**aggtggccagcggtg-5′*
_c-MYC_DS29^Hetero^	29	*5′-gcagagggcgtggg**ggaaaagaaaaaaga**-3′
		3′-cGtcTccCgcAccC**cCtTtTcTtTtTtCt**-Cy5-5′
_c-MYC_DS19	19	5′-gg**ggaaaagaaaaaaga**tc-3′
		3′-cc**ccttttcttttttct**ag-5′
_FXN_DS19	19	5′-gg**gaagaagaagaagaa**tc-3′
		3′-cc**cttcttcttcttctt**ag-5′
_c-MYC_DS19^Hetero^	19	5′-gg**ggaaaagaaaaaaga**tc-3′
		3′-cC**cCtTtTcTtTtTtCt**Ag-5′

LNA is indicated in capital letters and DNA is in small letters. Cy5 indicates the fluorophore used. DS, double strand; _c-MYC_DS45, _c-MYC_DS29, _c-MYC_DS19 and _FXN_DS19 are homo-duplex target sequences. Superscript ^Hetero^ indicates a hetero-duplex target sequence. The star (*) indicates the strand radiolabeled using [γ -^32^P] ATP isotope. The pyrimidine strand of _c-MYC_DS45 and _c-MYC_DS29 were radiolabeled. Nevertheless, for the _c-MYC_DS29^Hetero^, the purine strand was labeled due to that the pyrimidine strand was previously labeled with Cy5 fluorophore, and therefore unavailable for radiolabeling. Another difference between the target sequences is the size and the position of the TFO binding site. The size of _c-MYC_DS29 corresponds to the size of the WC29 (29-mer), and since the size is smaller compared to the _c-MYC_DS45, the TFO binding site is at the 3′-end and not in the center as for other target sequences. _c-MYC_DS19, _FXN_DS19, and _c-MYC_DS19^Hetero^ are target sequences used for simulations.

Based on the simulation results on the two systems (c-*MYC* and the *FXN*) we suggest that the effect of LNA on the DNA structure dominates the sequence specific effect. The quality and reliability of a molecular simulation depends on the quality of the force field used to describe the atomic interactions and on the reproducibility of the results, and validation depends on the availability of suitable experimental data. Here we used a state-of-the-art force field for nucleic acids^55^, with independently performed simulations, which exhibit stable and reproducible trends, and the agreement with EMSA data on c-*MYC* as validation.

### Stabilization of LNA-based triplex using TINA

**Figure 7:**
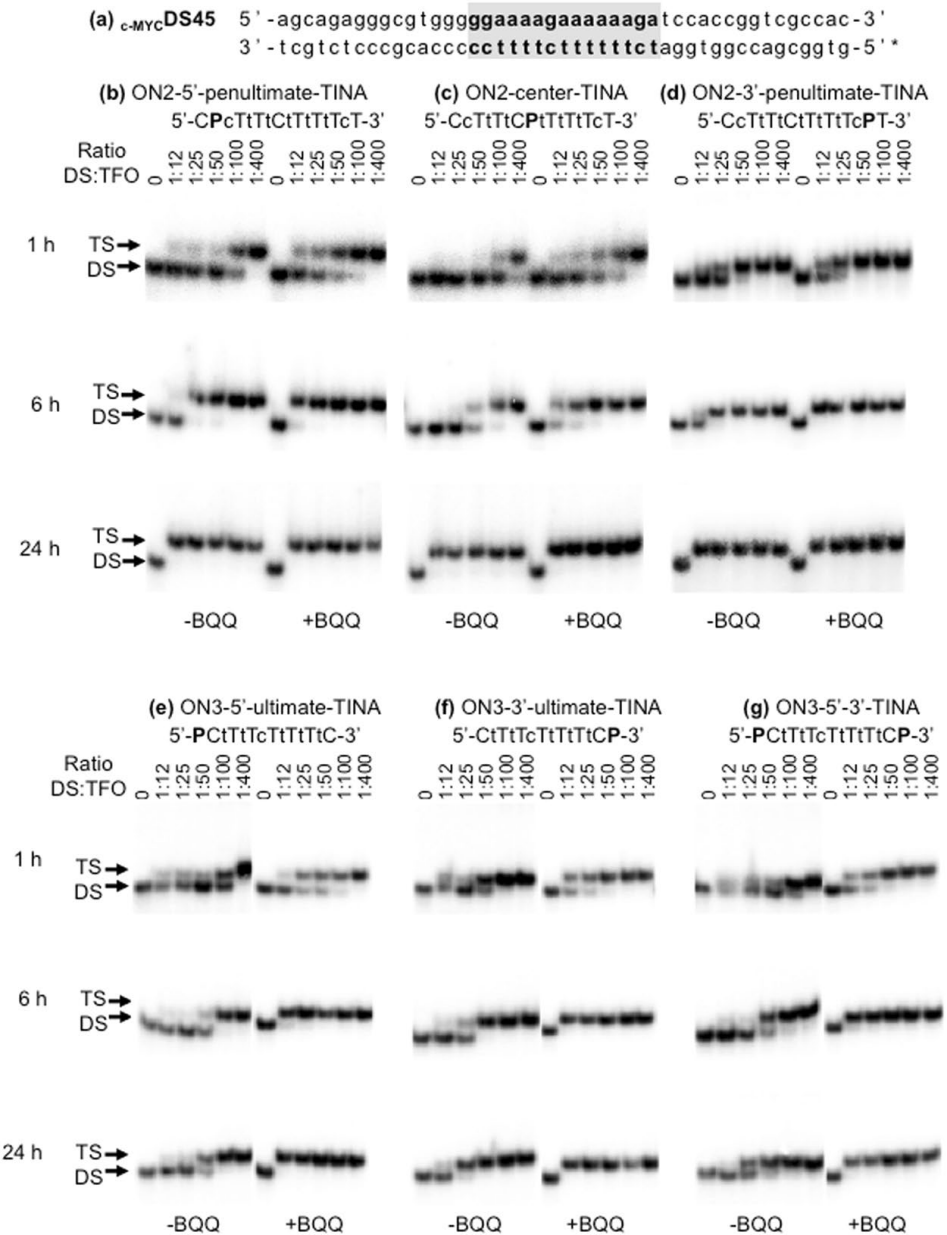
TFO binding of 13- and 15-mer ON sequences containing p-TINA (P) at different locations. (**a**) _c-MYC_DS45. (**b**,**c**,**d**,**e**,**f**,**g**) Electrophoretic mobility shift profile of _c-MYC_DS45 in the presence of different ONs, as indicated. Hybridization with ON in the absence (left side) and in the presence (right side) of BQQ carried out during 1, 6 and 24 h. Triplex structures are detected as slower migrating bands. DNA duplex and triplex complexes are indicated as DS and TS, respectively.

To further optimize binding of LNA-based TFOs, we synthesized several ONs (13 and 15-mers) carrying one or two TINA^39^ at different positions (Table 1). Initially, we compared three different 15-mer TFOs where TINA was placed at the penultimate 5′-end (ON2-5′-penultimate-TINA), in the middle (ON2-center-TINA) or at the penultimate 3′-end (ON2-3′-penultimate-TINA) (Table 1). All TFOs containing TINA show better triplex forming efficiency as compared to the control TFO (Fig. 3) and judged from the presence of shifted bands corresponding to triplex formation already after 1 h incubation (Fig. 7b–d). However, at the earlier time points (1 and 6 h) both ON2-5′-penultimate-TINA and ON2-3′-penultimate-TINA are more potent than ON2-center-TINA, which indicates that the effect of TINA at either end of the TFO is more significant. Furthermore, we detected essentially 100% triplex formation in the presence of ON2-3′-penultimate-TINA at lower TFO concentration and earlier time point (1 hour), as compared to ON2-5′-penultimate-TINA (Fig. 7b and d). These results indicate that stabilization of the 3′-end of a triplex may be more critical at the initial binding event than the corresponding 5′-end.

To further explore the stabilizing efficiency of TINA on LNA-based TFO binding, we synthesized shorter, 13-mer ONs. Again, the TFO having TINA at the penultimate 3′-end (ON3-3′-penultimate-TINA) was slightly more efficient than the one having TINA at the penultimate 5′-end (ON3-5′-penultimate-TINA) after 1 and 6 h of incubation in the absence of BQQ (Supplementary Fig. S4). Interestingly, a further increase in triplex-forming efficiency was observed when TINA was located at the ultimate 3′-end position of the TFO (Fig. 7e,f)), as compared to the penultimate 3′-end (Supplementary Fig. S4d). On the other hand, the ON3-5′-3′-TINA containing two TINAs (Fig. 7g), one in each end did not show improved binding as compared to the TFO containing TINA at the 3′-end (Fig. 7f). Obviously, the shorter TFO is less efficient, however; our analysis consistently shows that positioning TINA at the ultimate 3′-end (Fig. 7f) in parallel pyrimidine TFOs is most efficient.

### Effect of 5′-end Cy3-conjugation on triplex formation

Fluorescence labeling of ONs is commonly used to monitor target binding and also cell uptake and distribution. While conjugation of fluorescent probes to ONs is frequently used, this may also change their properties. Owing to the fact that most of the experimentally studied ONs are Cy3-conjugated, we compared the triplex-forming ability of the 5′-Cy3-conjugated LNA-ON (ON2-Cy3) (Table 1), previously described in the context of bisLNA^25^, to its cognate non-labeled ON (ON2-5′DNA) using the same dsDNA target (Table 2, _c-MYC_DS45).

**Figure 8:**
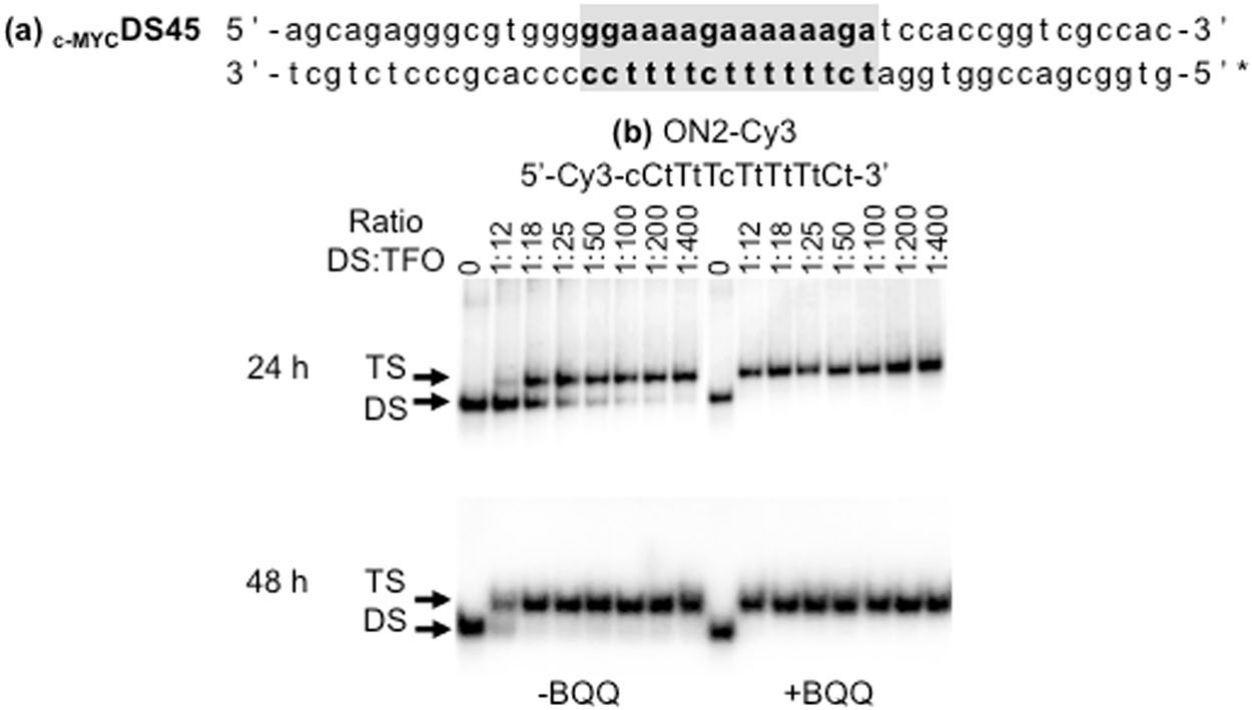
TFO binding of 15-mer ON sequences labeled with Cy3 fluorophore to a homo-duplex target sequence. (**a**) _c-MYC_DS45. (**b**) Electrophoretic mobility shift profile of _c-MYC_DS45 in the presence of ON2-Cy3. Hybridization with ON in the absence (left side) and in the presence (right side) of BQQ carried out during 24 and 48 h. Triplex structures are detected as slower migrating bands. DNA duplex and triplex complexes are indicated as DS and TS, respectively. LNA is indicated in capital letters and DNA is in small letters.

For the labeled TFO in the absence of BQQ, only a minor shifted band corresponding to triplex formation was first observed at 1:12 ratio of dsDNA:TFO (Fig. 8b, 24 h) and 90% triplex formation was reached at 1:400. Again, the triplex was further stabilized in the presence of BQQ. On the other hand, binding of the non-labeled TFO reached 40% at the lowest dsDNA:TFO ratio after 24 h (Fig. 2b) and essentially 100% triplex formation at 1:25 (Fig. 2b). This indicates that Cy3-conjugation at the 5′-end of a TFO has a negative effect on triplex formation. It is important to mention that both TFOs were equally efficient at low concentration (ratio 1:25) when incubation time was extended (48 h) (Fig. 3), which may suggest that Cy3-conjugation affects the rate of TFO binding to dsDNA rather than the stability of the end complex. We have previously reported bisLNA binding to dsDNA target in a supercoiled plasmid where the TFO-arm of the ON construct is conjugated to the Cy3-fluorophore^25^. Based on the current observation, we believe that TFO labeling may lead to underestimation of the binding affinity of bisLNA. Nevertheless, fluorescent compounds vary in their chemical structure and properties and to what extent conjugation of different probes may influence the hybridization of other classes of TFOs remains to be studied.

### Watson-Crick vs. Hoogsteen binding of LNA-ONs to short linear dsDNA

Based on previous studies on optimizing different elements in bisLNA^25, 26^, we decided to evaluate the effect of LNA substitution on triplex formation in the context of bisLNA. To this end, we compared how LNA-based ONs differ in WC and HG binding by targeting the dsDNA _c-MYC_DS45 sequence (Table 2) using the best-performing TFOs (based on previous experiments, Figs 3 and 5) and the WC29 ON. The binding experiments were performed with: 1. TFOs, 2. WC-ON, 3. TFO + WC-ON, and 4. bisLNA as control. The dsDNA target was incubated with high excess of LNA-ON (dsDNA:ON ratio, 1:400) and EMSA was used to analyze the different complexes. In parallel, binding of each ON was carried out in the presence of BQQ to identify complex(es) that contain a triplex structure. In all cases the binding reaction was followed at three different time points 1, 6 and 48 h.

**Figure 9:**
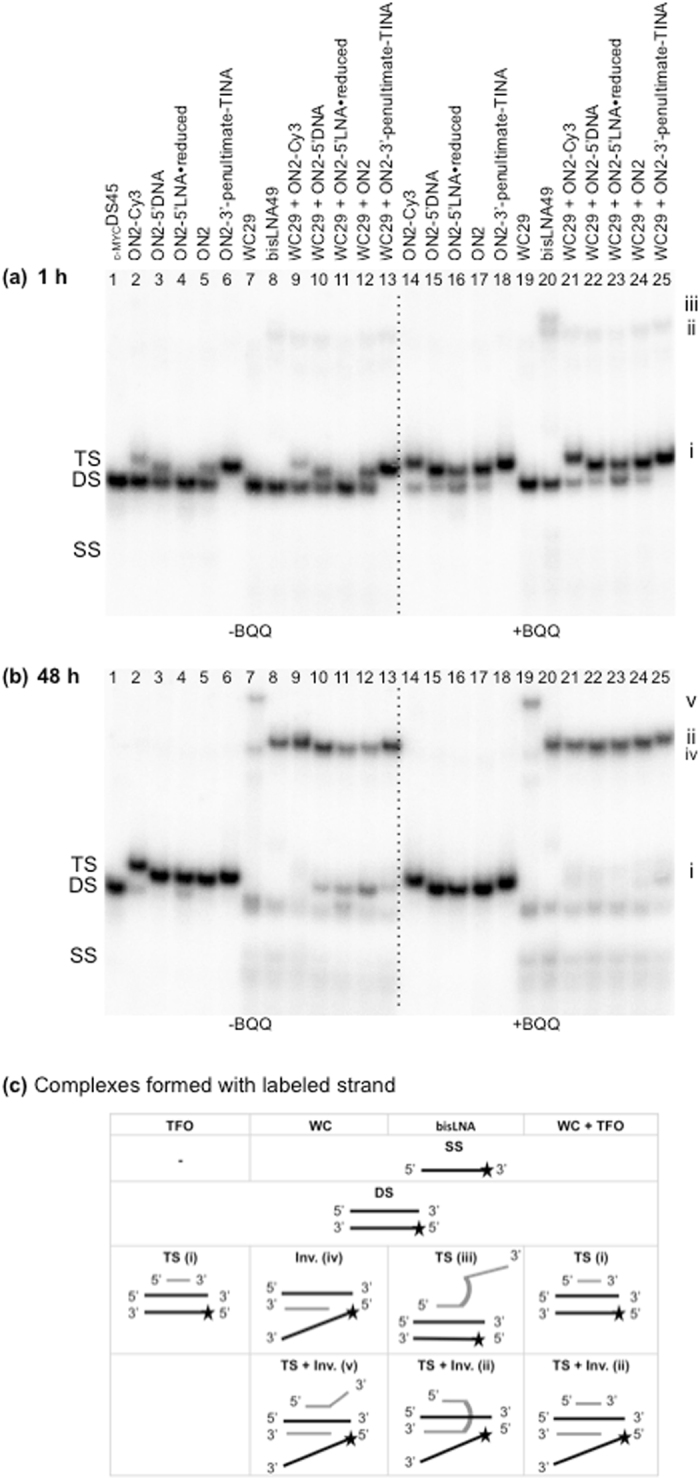
Comparison of TFO, WC and bisLNA constructs in shift assays. (**a**,**b**) Electrophoretic mobility shift profile of _c-MYC_DS45 in the presence of TFOs, WC, bisLNA and the combination of TFO and WC. Hybridizations with LNA-ONs at a concentration of 2 μM in the absence (left side) and in the presence (right side) of BQQ carried out during 1 and 48 h. Sequences are shown in Tables 1 and 2. Triplex structures are detected as slower migrating bands. Single stranded DNA, DNA duplex, triplex complexes and invasion are indicated as SS, DS, TS and Inv. respectively. (**c**) Schematic illustrations of the formed complexes are shown.

In the absence of BQQ, a clear shifted band appears as the evidence of triplex structure formation after shorter time of incubation (1 h) (Fig. 9a, complex i) for all TFOs except ON2-5′LNA ⦁ reduced. As expected, ON2-3′-penultimate-TINA reached essentially 100% triplex formation at this early time point. On the other hand, we did not detect any binding to the same dsDNA target in the presence of WC29 alone after 1 h of incubation (lanes 7 and 19) indicating that significant dsDNA invasion did not take place here. When we targeted the dsDNA with a combination of TFO and WC-ON, triplexes were formed (1 h) for all TFOs except ON2-5′LNA ⦁ reduced. Moreover, an additional complex was formed as judged from the appearance of a weak shifted gel band (lanes 9-13), which corresponds to the formation of a triplex-containing complex (ii) as confirmed by the enhanced intensity of the band in the presence of BQQ (lanes 21–25). Also, bisLNA binding resulted in a shifted band that corresponds to the same (slower) gel mobility and was further stabilized by BQQ (lanes 8 and 20). Interestingly, a second slower band was observed in the case of bisLNA in the presence of BQQ (lane 20). Here, we can state that both bisLNA-complexes are clearly stabilized by BQQ and hence they include a triplex structure (Fig. 9c, complex ii and iii).

Extended time of incubation of the different TFOs showed an increased binding and after 6 h all examined TFOs had reached a complete triplex formation in the presence of BQQ (Supplementary Fig. S5a). The intensity of the slow mobility band in the reactions including both TFO and WC-ON was also slightly stronger under these conditions. However, the major increase of band intensity was obtained in the bisLNA binding reaction, where both complexes (ii and iii) were stabilized by BQQ (Supplementary Fig. S5a, lane 20). In addition, a new band corresponding to a single strand appeared, which is in agreement with previous reports showing that comparable PNA-derived invasion of short linear dsDNA fragments can result in complete dissociation of the displayed pyrimidine strand^56^. Taken together, bisLNA binding of the linear dsDNA target and stabilization by BQQ results in one complex including both WC and HG binding (Fig. 9c, bisLNA-complex ii) and a second corresponding only to triplex formation with the TFO-arm of the bisLNA as shown in Fig. 9c (bisLNA-complex iii).

On the other hand, WC-ON binding occurred first after 48 h. As shown in Fig. 9b, an invasion complex was formed as judged from the presence of a new gel band (Fig. 9b, complex iv) and another band corresponding to the dissociated single strand (Fig. 9b, lanes 7 and 19). At the same time, a third and even slower mobility band appears, which is also stabilized by BQQ indicating formation of a triplex-containing WC-complex (Fig. 9c, complex v). As a matter of fact, WC29 is partially (10 nt) complementary to the TFO binding site and can form HG bonds and a triplex. Taken together, extended incubation time of WC29 leads to the formation of two complexes; the first being a dsDNA invasion and the second corresponds to both triplex and invasion (Fig. 9c, complex iv and v, respectively).

All the evaluated TFOs show nearly 100% TS formation after 48 h incubation, even in the absence of BQQ (Fig. 9b). Interestingly, the band corresponding to triplex, for each TFO, was converted to the slower mobility band (complex ii) when binding was carried out using a combination of TFO and WC-ON (Fig. 9b, lane 21–25). Again, this complex was further stabilized by BQQ, which confirms our model proposing a dual binding mode (WC and HG) of the dsDNA target (Fig. 9c, complex ii). To our knowledge, this is the first time a detailed and time-based analysis shows that a stable triplex can be converted to a triplex-invasion complex.

Finally, in contrast to what was seen at earlier time points, at 48 h bisLNA shows formation of only a single shifted band (Fig. 9b, lanes 8 and 20), and a displaced single strand. This pattern is in agreement with formation of a complex similar to that observed upon binding of TFO + WC29 (Fig. 9c, complex ii). Interestingly the second slower band (bisLNA-complex iii, lane 20), present at 1 and 6 h, disappeared, indicating again that the TFO arm of bisLNA binds first, followed by an invasion event forming a triplex-invasion complex. Our findings are in agreement with previously proposed mechanism for dsDNA-invading PNA and hence confirming a Hoogsteen-first model^12^. Detailed footprinting experiments indicated that PNA hybridization occurs by the initial binding of the TFO-arm forming HG hydrogen bonds with the duplex purine strand, followed by strand displacement and WC-arm hybridization of a second PNA oligomer^12^. However, it was important to investigate the mechanism for LNA ONs, since LNA and PNA exert different chemical properties and our experiments were carried out under intra-nuclear salt and pH conditions, and in contrast to PNA, the LNA ONs are devoid of lysine residues.

### LNA substitution affects DNA double helix conformation and subsequent TFO binding

It is well known that an LNA containing WC-ON is competitively superior to an unmodified DNA WC-ON regarding duplex formation^10, 11^. However, the implication of higher binding affinity in DNA:LNA duplex on triplex formation has not been assessed. Therefore, we designed a simplified model, in which TFO is bound to a “homo” dsDNA (without LNA) or a “hetero” dsDNA (with a DNA purine strand and an LNA/DNA alternating pyrimidine strand). This hetero-duplex target reflects the situation when the bisLNA has strand invaded into its dsDNA target.

**Figure 10:**
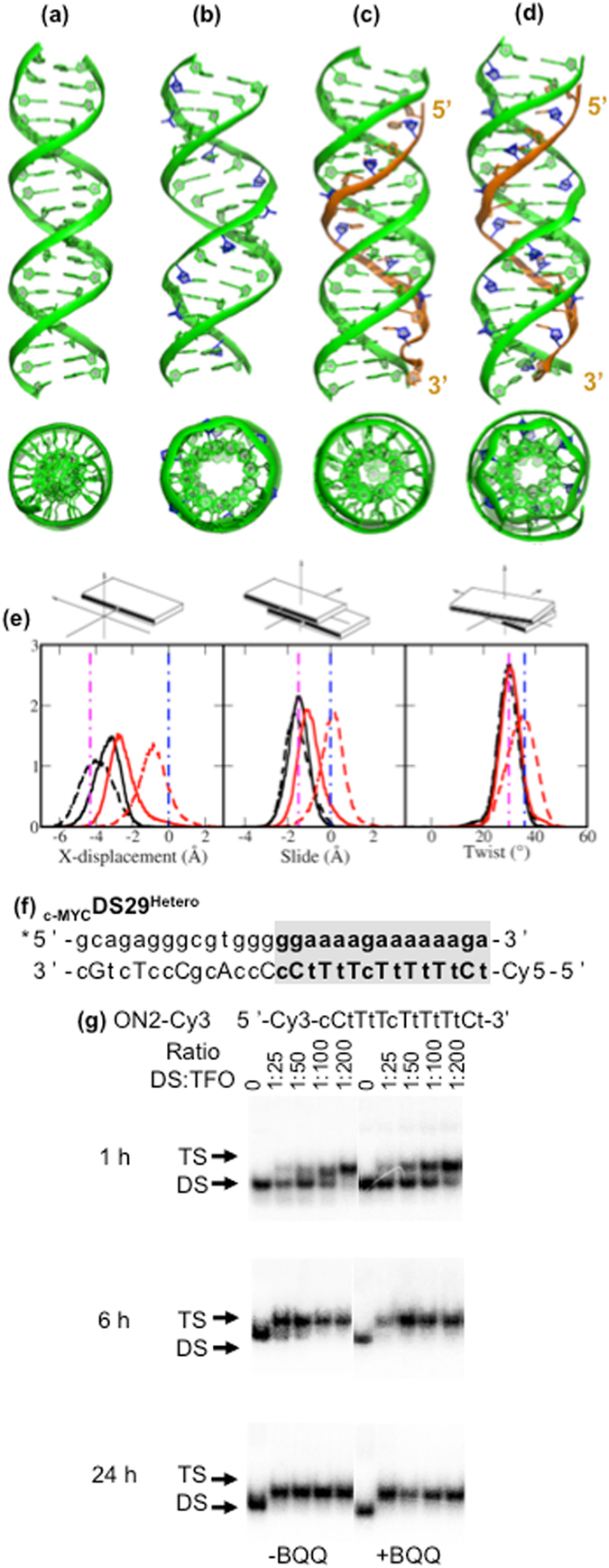
The average structures from MD simulations: (**a**) _c-MYC_DS19, (**b**) _c-MYC_DS19^Hetero^, (**c**) _c-MYC_DS19 ⦁ ON2-5′DNA and (**d**) _c-MYC_DS19^Hetero^ ⦁ ON2-5′DNA. The tertiary structures are shown in the front-view and 90°-rotated top-view. All LNA sugars are in blue and TFO strands in orange. In the top-view only the duplex strands are shown. Distribution of base-pair-step parameters (x-displacement, slide and twist): (**e**) For _c-MYC_DS19^Hetero^ and _c-MYC_DS19, as isolated duplex (dashed line) or bound to ON2-5′DNA (solid line). Curves in black are for _c-MYC_DS19^Hetero^ and in red for _c-MYC_DS19. The vertical dash-dotted lines represent the corresponding value of ideal A-DNA (in magenta) and B-DNA (in blue) duplex. The sketches explaining each base pair step are shown above each panel. TFO binding of 15-mer ON sequences labeled with Cy3 fluorophore to a hetero-duplex target sequence. (**f**) _c-MYC_DS29^Hetero^, (**g**) Electrophoretic mobility shift profile of _c-MYC_DS29^Hetero^ in the presence of ON. Hybridization with ON in the absence (left side) and in the presence (right side) of BQQ carried out during 1, 6 and 24 h. Triplex structures are detected as slower migrating bands. Single stranded DNA, DNA duplex and triplex complexes are indicated as DS and TS, respectively.

We simulated truncated _c-MYC_DS45 sequence where only the TFO binding site with two additional nucleotides at each end was kept (Table 2, _c-MYC_DS19 and _c-MYC_DS19^Hetero^) both in presence or absence of ON2-5′DNA. Duplexes _c-MYC_DS19 and _c-MYC_DS19^Hetero^ and the corresponding triplexes were stable in all simulations (Fig. 10a and b), with some fluctuation at the end of the TFO. Visually both duplexes showed minor stretching generating a wider major groove for TFO accommodation. From _c-MYC_DS19 to _c-MYC_DS19 ⦁ ON2-5′DNA, the duplex also underwent an apparent unwinding to enlarge the space (Fig. 10a and c). On the other hand, _c-MYC_DS19^Hetero^ originally had a larger diameter than _c-MYC_DS19, and no further unwinding was observed when TFO was bound (Fig. 10b and d).

No difference was observed in the sugar conformation of DNA nucleotides and in the major groove width between _c-MYC_DS19 and _c-MYC_DS19^Hetero^ (data not shown). There are however differences in x-displacement, slide and twist of base pair (Fig. 10e). In _c-MYC_DS19^Hetero^ the x-displacement and slide shifted toward more negative values (from −0.8 Å to −4.3 Å and from 0 Å to −1.6 Å, respectively) than in _c-MYC_DS19, and the twist shifted to lower values (from 36° to 30°). The main difference between A- and B-DNA duplexes is that while an ideal B-DNA is a perfectly straight helix, the A-DNA base pairs have negative x-displacement, slide, reduced twist, and increased inclination and roll^57, 58^. Our results show that _c-MYC_DS19 basically has a normal B-DNA conformation but a slightly negative x-displacement, whereas _c-MYC_DS19^Hetero^ has A-like x-displacement, slide and twist, but not enough inclination or roll to the helical axis to generate a full A-type conformation. Thus, the _c-MYC_DS19^Hetero^ conformation is between A and B, but close to A-type (a Low Inclination & Roll A-DNA: LirA DNA). The corresponding triplexes _c-MYC_DS19 ⦁ ON2-5′DNA and _c-MYC_DS19^Hetero^ ⦁ ON2-5′DNA however, show greater conformational similarity, with an average x-displacement of −2.5 Å and −3 Å, a slide of −1 Å and −1.5 Å, and a twist of 31° and 30°, respectively.

Comparing the conformations between duplex and TFO bound duplex (Fig. 10), it is clear that upon TFO binding _c-MYC_DS19 negatively shifts the slide and twist, which is consistent with the conformation reported for an antiparallel triplex with purine TFO^33^. However this is not the case for _c-MYC_DS19^Hetero^ where the duplex almost maintains the same conformation irrespective of the presence or absence of a TFO. Similar to the preorganization effect of LNA observed for the single strand TFO, the alternating DNA/LNA in the pyrimidine strand of the duplex promotes a conformation, where the third strand is more easily accommodated. This is of both practical and conceptual importance, since forming a hetero-duplex is advantageous for the hybridization of an HG-arm, which in turn would stabilize the invasion complex by forming a triplex structure.

These findings are valid for TFO binding of a longer hetero-duplex with the same TFO binding site (Table 2, _c-MYC_DS29^Hetero^) as analyzed using EMSA (Fig. 10f and g). When ON2-Cy3 was incubated we detected 50% triplex formation after 1 h (DS:TFO ratio 1:100) in the absence of BQQ and a complete binding at the lowest TFO concentration after 24 h (Fig. 10g). These findings demonstrate a major difference in binding efficiency of the same TFO to the hetero-duplex as compared to the dsDNA homo-duplex target (_c-MYC_DS45) (Fig. 8).

## Materials and Methods

### Oligonucleotides

Mixmer LNA/DNA ONs were synthesized by solid phase phosphoramidite chemistry on an automated DNA synthesizer in 1.0 mmol synthesis scale^18^. Purification to at least 85% purity of all modified ONs was performed by RP-HPLC or IE-HPLC, and the composition of all synthesized ONs was verified by MALDI-MS analysis recorded using 3-hydroxypicolinic acid as a matrix. The ONs and target sequences used here are presented in Tables 1 and 2, respectively. ON concentrations of stock solutions were determined using a Nanodrop spectrophotometer (Thermo Scientific).

### Preparation of ^32^P-labeled dsDNA target

The pyrimidine or purine strand of the target sequence was labeled using [γ-^32^P] ATP and T4 polynucleotide kinase (Fermentas) according to the manufacturer′s protocol, and then purified using QIAquick Nucleotide Removal Kit (Qiagen). The 5′-end labeled pyrimidine or purine ON was annealed with the unlabeled complementary strand at 1:1 ratio. The annealing was performed by heating for 5 min at 95 °C followed by 40 cycles during 1 min decreasing 1 grade per minute using a thermo cycler.

### Oligonucleotide hybridization

The double strand target (5 nM) was incubated with ON at different concentrations (0.06, 0.09, 0.125, 0.25, 0.5, 1 and 2 μM, corresponding to the following ratio of dsDNA target versus TFO, dsDNA:TFO, 1:12, 18, 25, 50, 100, 200 and 400 respectively). ONs were heated prior to hybridization during 5 minutes at 65 °C followed by cooling on ice. Hybridization was performed in intra-nuclear buffer (Tris-acetate 50 mM, pH 7.4, 120 mM KCl, 5 mM NaCl, 0.5 mM MgOAc) and in a total volume of 10 μl at 37 °C for 1, 6, 12, 24, 48 and 72 h in the absence or presence of the BQQ (1 μM).

### Electrophoretic Mobility Shift Assay (EMSA)

DNA complexes were analyzed using non-denaturing polyacrylamide gel electrophoresis 10% (29:1) in Tris acetate EDTA (TAE) buffer (1x, pH 7.4 supplemented with 0.5 mM MgOAc and 5 mM NaCl). The gels were run at 150 V, 200 mA during 4 to 5 h with circulation water-cooling and analyzed using a Molecular Imager FX. The intensity of the gel bands was quantified using Quantity One software (BioRad). All experiments were repeated three times.

### Molecular dynamics (MD) simulation

DNA molecules were simulated as single strand, duplex and triplex. The initial DNA duplex and triplex structures were built as canonical B-DNA duplex models or parallel DNA triplex fiber models using Maestro 9.3 (Schrödinger, LLC, New York, NY, 2013) and the w3DNA server^59^. Simulations were performed on graphical processing units with the program CHARMM^60^ and the CHARMM/OpenMM interface^61^, allowing production runs of 19-28 ns/day for our 65 000-atom systems. The CHARMM36 force field for nucleic acids^62^ and modified nucleotides^63, 64^ was used for the DNA molecules, and the TIP3P model^65^ for water molecules. Cytosines were protonated in the TFO strand. Before solvation the structures were energy-minimized in 500 steps using the Adopted-Basis Newton-Raphson method, with harmonic restraints (with a force constant of 20 kcal/mol/Å^2^) on backbone atoms. All structures were solvated in a cubic water box, with the shortest distance between box edge and solute of at least 8 Å and periodic boundary conditions were applied. The systems were neutralized by adding sodium ions, and 0.15 M NaCl was added in some cases; this results in Na^+^ concentrations between 0.1 M and 0.27 M (Table 3). The particle mesh Ewald method^66^ was applied for long range electrostatic interactions, with a direct space cutoff of 9 Å, and a switch (vswitch) over the range 8–9 Å was used for van der Waals interactions. The simulations were performed in the NVT ensemble using Langevin dynamics with a friction coefficient of 5 ps^–1^. The leap-frog integrator was used with a 2 fs time step. Bonds involving hydrogen atoms were constrained using the SHAKE algorithm^67^. The systems were equilibrated by running first a 10 ns simulation at 298 K, in which harmonic restraints were applied to the N1-N3 distance for the WC base pairs and to the N7-N3 distance for HG base pairs, with a restraint force constant of 10 kcal/mol/Å2 on end-WC base pairs and 5 kcal/mol/Å^2^ on other pairs. This was followed by another 20 ns equilibration where all restraints were released except for the last WC base pairs and the last two HG base pairs in each end. The production run was carried out for at least 120 ns, with only end-WC base pairs restrained. Table 3 summarizes all the performed simulations. All the simulated TFOs are homopyrimidine ONs and the targeted duplexes composed of homopurine ⦁ homopyrimidine ON sequences.

**Table 3:** The systems used in Molecular dynamics (MD) simulations.

Model name^1^	Box edge (Å)	[Na^+^] | simulation time
**Single strand**		
ON1	70	0.12 M|120 ns
ON2-5′DNA		
		0.20 M|120 ns
**Duplex**		
_c-MYC_DS19	83	0.10 M|120 ns
_c-MYC_DS19^Hetero^		0.25 M|140 ns
**Triplex**		
_c-MYC_DS19 ⦁ ON2-5′DNA	85	0.12 M|120 ns, 140 ns
_c-MYC_DS19^Hetero^ ⦁ ON2-5′DNA		0.27 M|220 ns
_c-MYC_DS19 ⦁ ON2-3′LNA ⦁ reduced	85	0.27 M|200 ns, 140 ns
_c-MYC_DS19 ⦁ ON2-5′LNA ⦁ reduced		
_FXN_DS19 ⦁ ON4-3′LNA ⦁ reduced	85	0.27 M|200 ns
_FXN_DS19 ⦁ ON4-5′LNA ⦁ reduced		
_c-MYC_DS19 ⦁ ON3-3′LNA ⦁ reduced-c/t	77	0.15 M|140 ns
_c-MYC_DS19 ⦁ ON3-5′LNA ⦁ reduced-c/t		

^1^ONs and target sequences (DS) are shown in Tables 1 and 2, respectively. The triplex is represented by duplex ⦁ ON.

### Structural analysis

MD snapshots, saved every 40 ps, were analyzed using CHARMM and Curves+^68^. To check the maintenance of base pairs in the duplex and triplex, the N1-N3 distances for WC base pairs and N7-N3 for HG base pairs, were monitored. A distance shorter than 3.5 Å indicates that a hydrogen bond is formed between the heavy atoms and the bases are considered to be paired. The conformation was characterized using the glycosidic torsion, sugar pucker, base pair step parameters, and major/minor groove dimension. If not otherwise specified, the analysis was performed excluding the last two nucleotides in each strand.

The glycosidic torsion (χ) is defined by the dihedral O4′-C1′-N1-C2 (pyrimidine) or O4′-C1′-N9-C4 (purine), and its main conformations are denoted as *anti* (170° < χ < 320°, where χ < 220° is low *anti* and >270° is high *anti*) and *syn* (30° < χ < 90°). The sugar pucker is defined by the pseudorotation phase angle (*P*), which is a combination of five ring torsions, and it is denoted as *north* (−90° < *P* ≤ 90°) and *south* (90° < P ≤ 270°).

## Conclusions

The combination of electrophoretic mobility shift assay (EMSA) hybridization analysis and atomistic simulations allows us to better understand the Watson-Crick (WC) and Hoogsteen (HG) binding of LNA substituted ONs. We found that the inclusion of LNA in both TFO and WC ONs enhances triplex formation and affects conformational flexibility of not only single strand, but also of duplex and triplex structures.

LNA-containing single strand TFOs, are conformationally pre-organized for major groove binding and their binding to a dsDNA target was clearly detectable, whereas the corresponding, non-modified DNA ON failed to bind and form triplex. Reducing the LNA content in the 3′-end impaired hybridization as compared to reduction in the 5′-end, as observed in both EMSA and modeling. Similarly, inclusion of the triplex-intercalator TINA in TFOs potently stabilizes triplex formation, with 3′-end TINA being more efficient than 5′-end inclusion and much more efficient than TINA being centered in the TFO.

There are at least two aspects to be considered, namely the rates of association and dissociation of TFOs. It is well known that LNA modification in TFOs decreases mainly the dissociation rate^48, 69^. Based on the nucleation-zippering model^70^, substitution of LNA by six non-modified deoxynucleotides at one end of the TFO would be expected to affect the nucleation step. Similarly, this substitution could also impact on the dissociation rate of the TFO. Our results indicate that the triplex destabilization effect is preferentially detected when this substitution takes place at the 3′-end of the TFO. In analogy, triplex formation at lower pH, presumably stabilizing the formed triplex, affects binding of the 3′-LNA-reduced TFO to a greater extent than the corresponding 5′-LNA-reduced TFO (unpublished).

Furthermore, this is the first report on triplex-specific stabilization of LNA-containing TFOs by the triplex-intercalator BQQ. In all contexts BQQ improves triplex formation. Moreover, LNA-substitutions in the WC pyrimidine strand alter the duplex structure, generating a Low Inclination & Roll A-DNA (LirA DNA) conformation, which forms with base pairs remaining almost perpendicular to the helical axis but with negative slide and x-displacement, and reduced twist. This conformation is observed after the TFO is bound to both DNA homo-duplex and hetero-duplex, where one of the strands is a DNA-LNA mixmer. Such a hetero-duplex is formed when so-called bisLNA^25, 26^ invades into a homo-duplex DNA. Indeed EMSA experiments show that a hetero-duplex target forms a triplex faster and at a lower TFO concentration than the corresponding homo-duplex.

The WC-ON and bisLNA invasion of dsDNA shows formation of different complexes over time. Based on these observations we propose the following mechanism for bisLNA binding: The TFO-arm first binds to the target dsDNA allowing HG base pairs to form a triplex. The target dsDNA is invaded through DNA-LNA hybridization by competitively forming new WC base pairs. The latter causes the release of the un-bound DNA strand, sometimes referred to be the ‘displacement loop’, or D-loop. During this process the TFO-arm is rearranged and thereafter reforming HG base pairs, but now with the hetero-duplex, a conformationally more favorable hybridization as compared to the initial binding to duplex-DNA.

Our findings can help simplify the design of LNA containing anti-gene ONs, including TFOs and bisLNAs, regarding the number and location of LNA substitutions in parallel pyrimidine TFOs, and the conjugation of intercalating compounds or fluorescent probes. Mainly, for TFOs, it is advantageous (i) to include at least 30% of LNA substitution at the 3′-end. (ii) to introduce TINA at the 3′-end, as well. (iii) to take into consideration that Cy3-conjugation of the 5′-end of TFO can interfere with binding. Also, we provide a theoretical basis for understanding the hybridization process of TFOs, both for DNA homo- and hetero-duplex targets, which can potentially further advance the use of triplex-based constructs in future cell and *in vivo* applications.

## Electronic supplementary material

Supplementary information
