# Supplementary material for: LNA effects on DNA binding and conformation: from single strand to duplex and triplex structures
Source: Sci Rep. 2017 Sep 8;7:11043. doi: 10.1038/s41598-017-09147-8 (PMC5591256; doi:10.1038/s41598-017-09147-8)
Supplement: Supplementary file 1 — Supplementary information [file 41598_2017_9147_MOESM1_ESM.pdf]

Supplementary information

LNA effects on DNA binding and conformation: from single strand to duplex and triplex structures

Y. Vladimir Pabon-Martinez[1,+], You Xu[2,+], Alessandra Villa[2], Karin E. Lundin[1], Sylvain Geny[1], Chi-Hung Nguyen[3], Erik B. Pedersen[4], Per T. Jørgensen[4], Jesper Wengel[4], Lennart Nilsson[2], C. I. Edvard Smith[1] and Rula Zain[1, 5,*]

Contents:

Figure S1. TFO binding of 15-mer ON sequence.
Figure S2. The WC and HG hydrogen bond
Figure S3. Distribution of glycosidic torsion (X) and ribose pseudorotation (P) of nts in TFO.
Figure S4. Effect of LNA position on triplex formation in $_{FXN}$DS19•ON4-3'LNA•reduced and $_{FXN}$DS19•ON4-5'LNA•reduced.
Figure S5. TFO binding of 13-mer ON sequences containing p-TINA (P) at different locations.
Figure S6. Comparison of TFO, WC and bisLNA constructs in shift assays.

Figure S1.

(a) $_{c\text{-}MYC}$DS45

5’-agcagagggcgtggg**ggaaaagaaaaaaga**tccaccggtcgccac-3’
3’-tcgtctcccgcaccc**ccttttcttttttct**aggtggccagcggtg-5’*

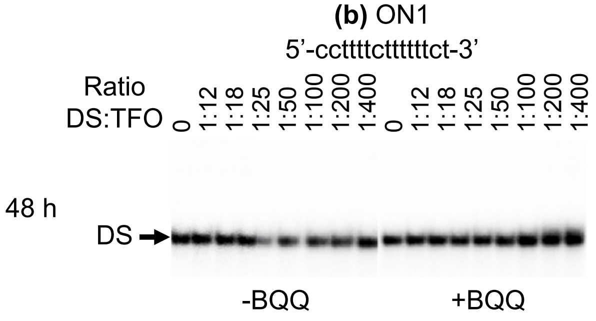

Figure S1. TFO binding of 15-mer ON sequence. (a) $_{c\text{-}MYC}$DS45, (b) Electrophoretic mobility shift profile of $_{c\text{-}MYC}$DS45 in the presence of a DNA ON. Hybridization with ON in the absence (left side) and in the presence (right side) of BQQ carried out during 48 h (24 and 72 h, data not shown). DNA duplex is indicated as DS.

Figure S2.

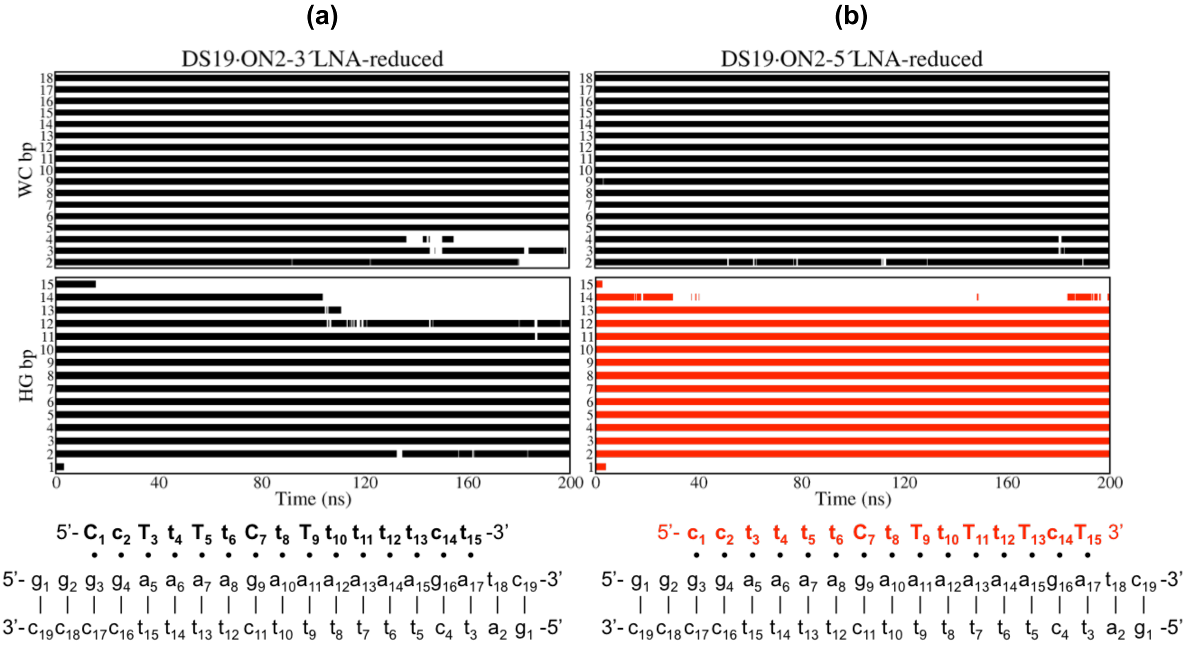

Figure S2. The WC and HG hydrogen bond as the function of simulation time. WC bp and HG bp hydrogen bond and the corresponding sequences of triplex. (Panel a) $_{\text{c-MYC}}$DS19•ON3-3’LNA•reduced and (Panel b) $_{\text{c-MYC}}$DS19•ON3-5’LNA•reduced; ON3-3’LNA-reduced is in black and ON3-3’LNA-reduced is in red. Blank spaces correspond to the loss of the hydrogen bonds.

Figure S3.

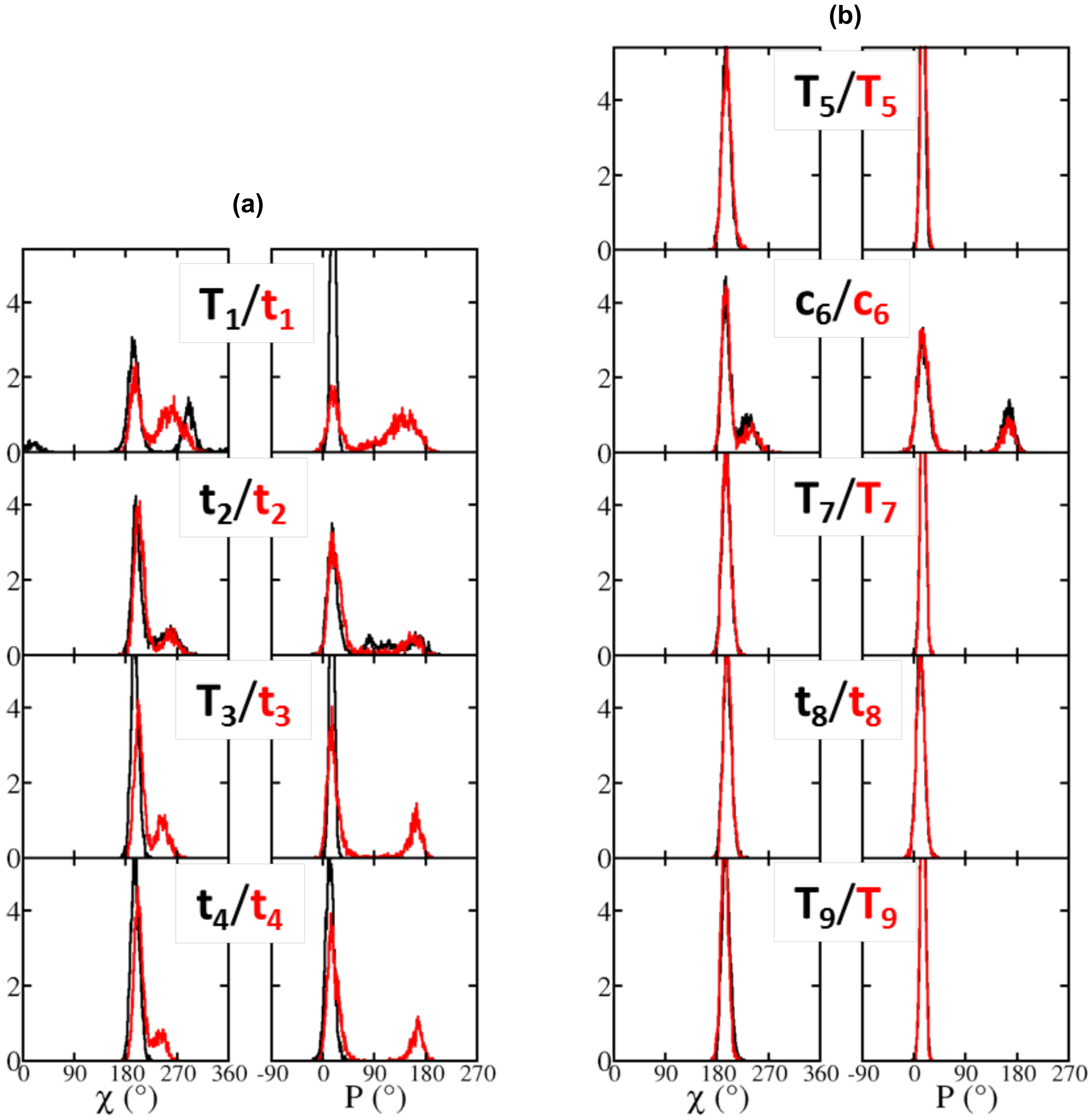

Figure S3. Distribution of glycosidic torsion (X) and ribose pseudorotation (P) of nts in TFO. The first four nts at 5'-end (panel a) and middle five nts (panel b) for triplex $_{c\text{-}MYC}$DS19•ON3-3'LNA•reduced-c>t (black) and $_{c\text{-}MYC}$DS19•ON3-5'LNA•reduced-c>t (red) are showing.

Figure S4.

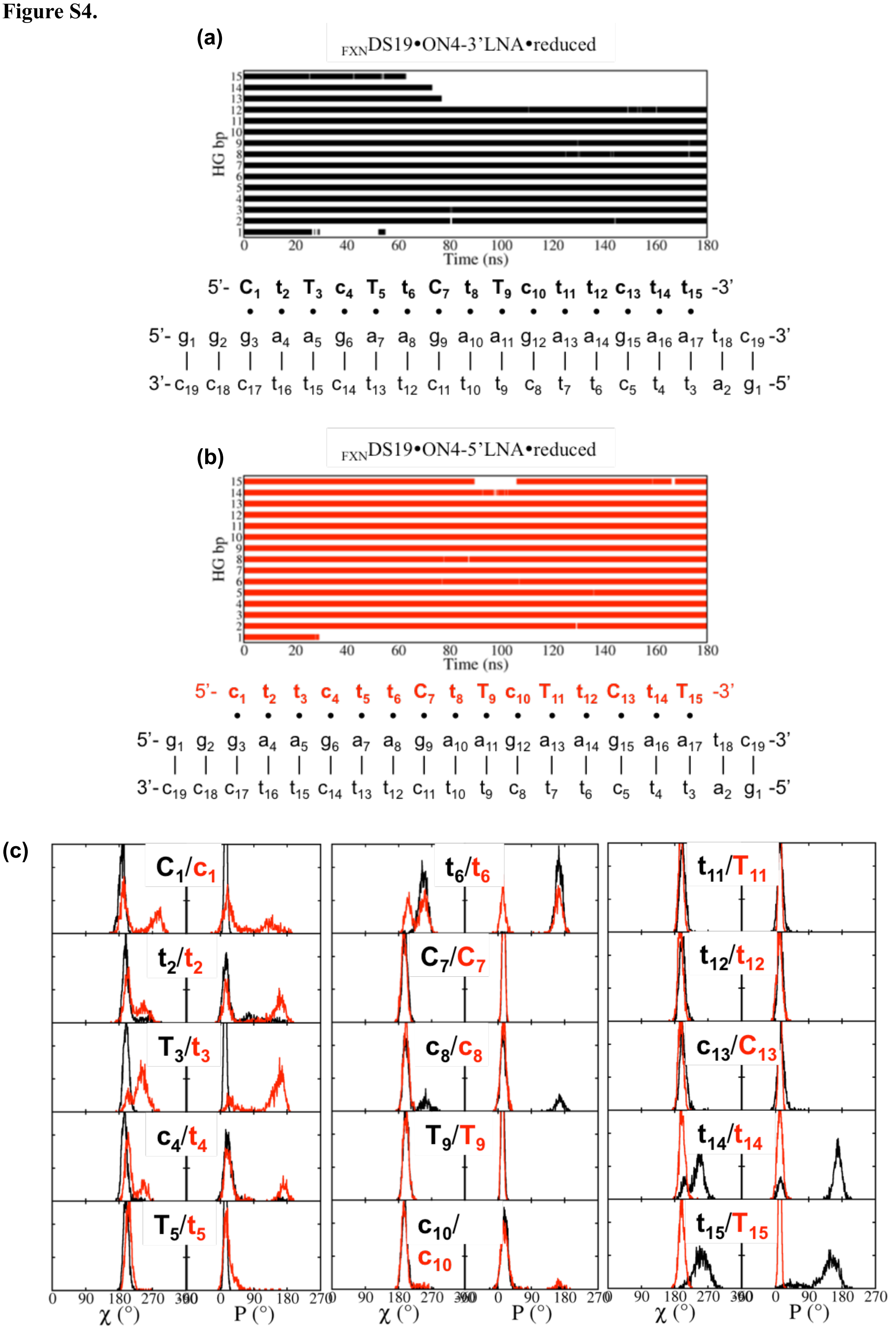

Figure S4. Effect of LNA position on triplex formation in $_{FXN}$DS19•ON4-3'LNA•reduced and $_{FXN}$DS19•ON4-5'LNA•reduced. HG hydrogen bonds as function of time in (Panel a) Triplex $_{FXN}$DS19•ON4-3'LNA•reduced, and (Panel b) Triplex $_{FXN}$DS19•ON4-5'LNA•reduced; the blank spaces correspond to the loss of the hydrogen bonds. (Panel c) Distributions of χ and P for ON4-3'LNA•reduced in black and ON4-5'LNA•reduced in red. The distributions were accumulated from the first 70 ns, during which no base pair opening happened at the 3'-end.

Figure S5.

(a) $_{c\text{-}MYC}$DS45

5'-agcagagggcgtggg**ggaaaagaaaaaaga**tccaccggtcgccac-3'
3'-tcgtctcccgcaccc**ccttttcttttttct**aggtggccagcggtg-5'*

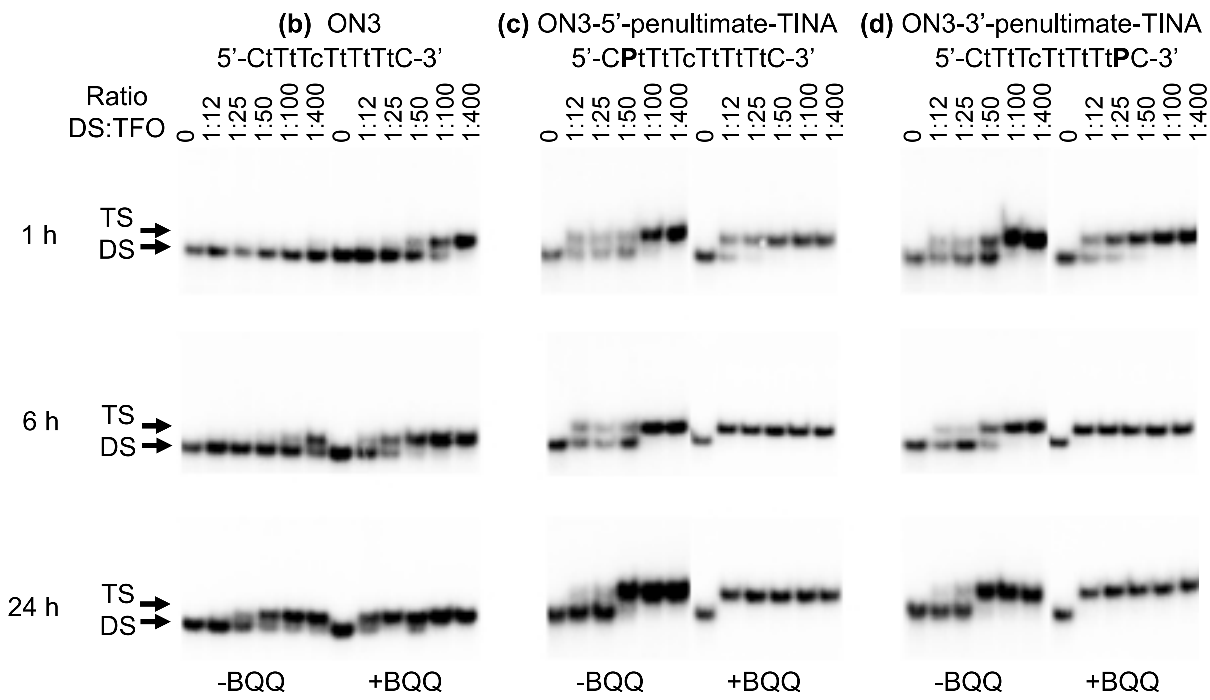

Figure S5. TFO binding of 13-mer ON sequences containing p-TINA (P) at different locations. (a) $_{c\text{-}MYC}$DS45, (b, c, d) Electrophoretic mobility shift profile of $_{c\text{-}MYC}$DS45 in the presence of different ONs. Hybridization with ON in the absence (left side) and in the presence (right side) of BQQ carried out during 1, 6 and 24 h. Triplex structures are detected as slower migrating bands. DNA duplex and triplex complexes are indicated as DS and TS, respectively.

Figure S6.

(a) 6 h

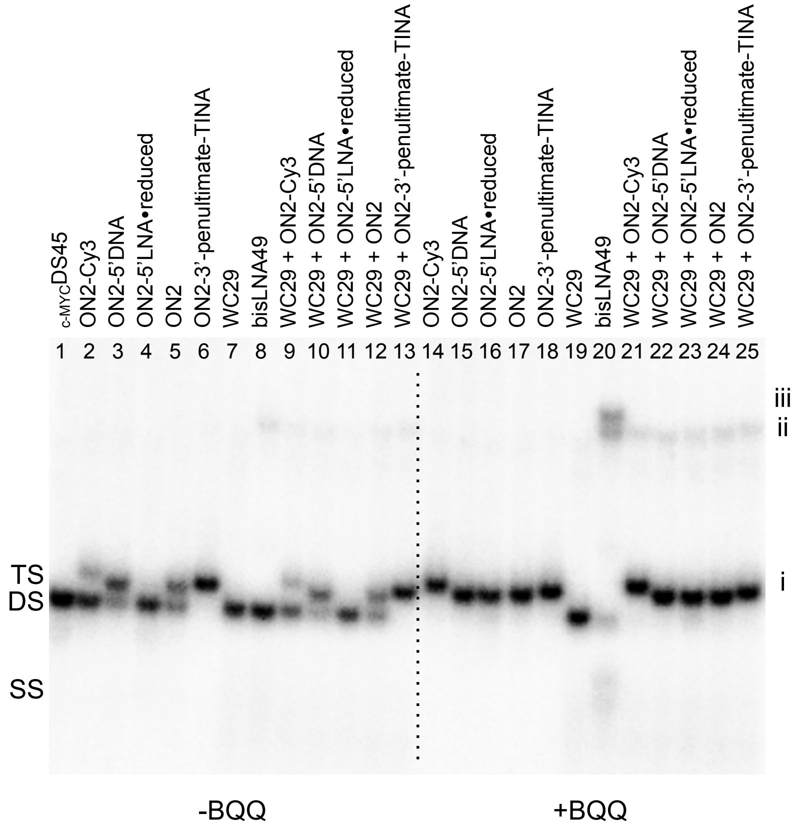

Figure S6. Comparison of TFO, WC and bisLNA constructs in shift assays. (a) Electrophoretic mobility shift profile of $_{c\text{-}MYC}$DS45 using TFO, WC, bisLNA and the combination of TFO and WC. Hybridization with LNA-ONs in the absence (left side) and in the presence (right side) of BQQ carried out during 6 h. Sequences are shown in Table 1 and 2. Triplex structures are detected as slower migrating bands. Single stranded DNA, DNA duplex, triplex complexes and invasion are indicated as SS, DS, TS and Inv. respectively.
